# Recruitment for Occupational Research: Using Injured Workers as the Point of Entry into Workplaces

**DOI:** 10.1371/journal.pone.0068354

**Published:** 2013-06-27

**Authors:** Mieke Koehoorn, Catherine M. Trask, Kay Teschke

**Affiliations:** 1 School of Population and Public Health, University of British Columbia, Vancouver, British Columbia, Canada; 2 Canadian Centre for Health and Safety in Agriculture, University of Saskatchewan, Saskatoon, Saskatchewan, Canada; Universität Bochum, Germany

## Abstract

**Objective:**

To investigate the feasibility, costs and sample representativeness of a recruitment method that used workers with back injuries as the point of entry into diverse working environments.

**Methods:**

Workers' compensation claims were used to randomly sample workers from five heavy industries and to recruit their employers for ergonomic assessments of the injured worker and up to 2 co-workers.

**Results:**

The final study sample included 54 workers from the workers’ compensation registry and 72 co-workers. This sample of 126 workers was based on an initial random sample of 822 workers with a compensation claim, or a ratio of 1 recruited worker to approximately 7 sampled workers. The average recruitment cost was CND$262/injured worker and CND$240/participating worksite including co-workers. The sample was representative of the heavy industry workforce, and was successful in recruiting the self-employed (8.2%), workers from small employers (<20 workers, 38.7%), and workers from diverse working environments (49 worksites, 29 worksite types, and 51 occupations).

**Conclusions:**

The recruitment rate was low but the cost per participant reasonable and the sample representative of workers in small worksites. Small worksites represent a significant portion of the workforce but are typically underrepresented in occupational research despite having distinct working conditions, exposures and health risks worthy of investigation.

## Introduction

Studies of occupational exposures and health risks present researchers with a number of practical methodological challenges including gaining access to workers and workplaces to collect data, and ensuring representation of the workforce across jobs, worksites, and industries. Literature on recruitment methods in occupational research is limited [1]–[5]. Traditionally, occupational epidemiology or exposure assessment research has relied on access to workers via large worksites [e.g. 6], employers [e.g. 7], industry associations [e.g. 8] or unions [e.g. 9]. These conventional recruitment approaches may under-represent segments of the workforce, especially workers in small worksites (i.e. those with less than 20 employees) [10], [11], and transient workers or work locations such as in construction [1].

In most industrialized and high-income countries, small workplaces represent a significant portion of worksites and employ significant segments of the workforce. In Canada, over 85% of private sector worksites employ less than 20 workers (including the self-employed) [12]. Excluding the self-employed, over 25% of the workforce work in small worksites [13]. In European countries, over 90% of worksites are categorized as small (<10 persons) and employ 30% of the workforce [14]. Small worksites represent environments with different working conditions, exposures, and health risks than larger companies that warrant inclusion in occupational health research [15]–[19], but that may be hard to reach [20]. Diversifying study samples to be population-based and representative of all working environments may improve the chances of identifying exposures, health risks and exposure-response relationships, and broaden applicability of findings across the working population, including small worksites.

The purpose of this paper was to evaluate using injured workers as the initial point of contact for recruitment for a population-based, epidemiological study of occupational exposure-response relationships. This method was developed as part of a back injury program of research that included on-site ergonomic assessments to evaluate the relationship between physical exposures and risk of back injury in heavy industry, although the recruitment method has relevance for other occupational epidemiology studies. The evaluation included an investigation of the feasibility, costs and sample representativeness of the recruitment method. Feasibility was assessed relative to recruitment targets and participation rates. Research costs have practical implications for study design, and are a vital input for evaluating recruitment and exposure assessment methods for representativeness and diversity in study samples [21]–[24]. Finally, sample representation was evaluated relative to overall workforce characteristics, in particular the ability to recruit workers representative of small worksites (<20 employees) that are under-represented in occupational health research and may be harder to reach using traditional recruitment strategies that have relied on large employers or worker associations.

## Materials and Methods

### Ethics Statement

The Researchers and WorkSafeBC (the provincial workers’ compensation agency) signed a data access agreement governing the use of compensation claim data for research purposes (DAR#03-032). The Behavioural Research Ethics Board, University of British Columbia (Certificate#B03-0644) approved the project procedures. According to privacy legislation governing research in this jurisdiction, individuals in the workers’ compensation database were requested to provide verbal permission over the telephone to a WorkSafeBC representative to release their contact information to researchers for research purposes [25]. Among workers who agreed to release their contact information, informed written consent was obtained for those recruited by the researchers for participation in the study. Informed written consent was also obtained for co-workers recruited into the study. Documentation was maintained by the researchers of those who were contacted, number of times contact was attempted, number recruited, and number of refusals/reasons for refusal to participate.

### List of Potential Participants

Gaining access to workers representative of the broader workforce is dependent upon access to a population-based list of workers. In Canada, a complete list is only maintained by the income tax agency, but is not accessible for research purposes. An alternate source is workers’ compensation records. Though these records include injured workers only, they are available for research purposes, and weight recruitment to worksites with higher injury or illness rates, a reasonable emphasis for occupational health research. For this study, injured workers with a time-loss, workers’ compensation claim for back strain in 2001 were identified as potential study participants. The year 2001 was chosen in order to increase the likelihood that workers would be injury and disability ‘free’ (i.e. recovered from previous back injury and returned-to-work) at the time of study recruitment in 2005 and 2006 [26]. The workers’ compensation system in the Canadian province of British Columbia provides coverage to over 93% of worksites and 92% of these are small worksites [27], [28]. Injured workers were randomly selected from five target heavy industries with a high rate of back strain claims: forestry, wood and paper products, warehousing, construction and transportation.

### Initial Contact – Release of Contact Information

A representative of the workers’ compensation agency contacted the randomly selected workers between September 2005 and April 2006. Those contacted were provided with a scripted introduction to the study, written by the researchers and approved by the ethics board, and were asked to provide verbal permission to release their contact information to the researchers. The goal was 50 participants, 10 in each of the five target industries.

### Researcher Contact – Eligibility/Consent to Participate

All individuals who agreed to release their contact information were sent a letter from the researchers describing the study and inviting their participation in on-site ergonomic assessments during regular work shifts. Exposure assessment methods and their costs are described elsewhere [24], [29]. Workers were contacted by a researcher approximately two to four weeks after the letter was mailed to answer questions, assess eligibility, invite participation in the study and provide written consent to participate. Workers who had retired since their claim, were on work disability, were performing modified duties, had left the workforce, or were no longer working in the five target industries were excluded. Workers who released their contact information but were not reached on the first telephone call received a minimum of four follow-up calls during the week including the weekend. Efforts were made to update contact information by searching resources such as on-line directories.

### Worksite Recruitment

The next step was obtaining employer consent to conduct on-site exposure assessments. Consenting workers were given the option of contacting their employer directly with the study letter of invitation or have the researchers contact their employer. Employers were offered a study information package and a chance to meet with researchers at the worksite for a study presentation. Benefits of participation were emphasized, including the value to worker and union relations of supporting safety research and receiving a copy of the study report on ergonomic risk factors. Consenting workers whose employers refused to participate were not included in the study sample.

### Co-worker Recruitment

At participating worksites, two co-workers in production jobs were sought for recruitment. During worksite visits, co-workers were recruited by the initial participating worker, their immediate supervisor, or the researchers. Potential co-worker participants were given a letter describing the study and providing researchers’ contact information for those who wanted to participate. Workers and/or worksites that refused to recruit co-workers, were too small to have additional ‘shop floor’ workers, or were unsuccessful in recruiting co-workers remained in the study sample. The goal was to recruit two co-workers per injured worker, i.e. 100 co-workers, for a total study sample of 150 participants.

### Recruitment Costs

Worker Recruitment Costs: Costs for the workers’ compensation agency participation in recruitment were based on an average of four calls per worker and approximately five minutes per call at an hourly rate of CDN$35.00 per hour. Development, printing, and postage costs for the invitation letters were based on an average of CDN$5.87 per individual. Costs for researcher time were based on an average of 55 minutes per worker including repeated calls and documentation, at a rate of CDN$20.00 per hour.

Worksite Recruitment Costs: Costs for employer information packages were based on an average of CDN$3.10 per package. Costs for phone calls to employers were based on an average of 85 minutes, including repeated calls and documentation, at a rate of CDN$20.00 per hour**.** Costs for worksite recruitment visits, including researcher travel time, were based on an average of 2.5 hours per worksite at a rate of CDN$20.00 per hour, an average distance of 100 km per round trip at CDN$0.40/km, and average additional transportation costs (e.g. highway or ferry tolls) of CDN$7.74 per trip.

### Representativeness

Worker and worksite characteristics were collected by questionnaire as part of the on-site ergonomic assessment protocol [24], [29]. The final study sample was compared to provincial workforce statistics on sex and age characteristics, and on the distribution of worksites by workplace size.

## Results

### Feasibility

The workers’ compensation agency identified a random sample of 822 workers with an accepted, short-term disability claim for back strain in 2001 in one of the five target industries and living in the study area at the time of the injury. An agency representative was able to successfully contact 358 of the 822 workers (Table 1). Of these, 189 agreed to release their contact information to researchers and 155 contacted by the researchers after an average of three phone calls (range 1 to 8). Of those contacted, 105 were eligible. Excluded participants tended to be out of the workforce or working in a different (non-study) industry. There was no statistical difference (p>0.05) in the proportion of ineligible workers by industry. Of the eligible workers, 74 consented to participate. There was no statistical difference (p>0.05) in the worker refusal rates by industry.

**Table 1:** Summary of Contact and Recruitment Steps.

**Random Sample of Eligible Workers**		
n = 822		
↓		
**Contacted by Compensation System**	**Not Contacted**	
n = 358 (44%)	n = 464 (56%)	
↓	↓	
↓	**Reasons for Non Contact:**	
↓	Incorrect Phone Number	n = 156 (34%)
↓	Unreturned Phone Call	n = 133 (29%)
↓	Other	n = 175 (37%)
↓		
**Worker Consented to Release of Contact Information to Researchers**	**Worker Declined**	
n = 189 (53%)	n = 169 (47%)	
↓		
↓		
**Contacted by Researchers**	**Not Contacted**	
n = 155 (82%)	n = 34 (18%)	
↓	↓	
↓	**Reasons for Non Contact:**	
↓	Incorrect Phone Number	n = 19 (56%)
↓	Unreturned Phone Call	n = 15 (44%)
↓		
**Eligible for Study**	**Ineligible**	
n = 105 (68%)	n = 50 (32%)	
↓	↓	
↓	**Reasons for Ineligibility:**	
↓	Wrong Industry	n = 17 (35%)
↓	Not Working	n = 14 (29%)
↓	Retired	n = 12 (24%)
↓	Other	n = 6 (12%)
↓		
**Worker Consented to Participate in Study**	**Worker Declined**	
n = 74 (70%)	n = 31 (30%)	
↓	↓	
↓	**Reasons for Declining to Participate:**	
↓	No Reason	n = 8 (26%)
↓	Annoyance	n = 5 (16%)
↓	Avoid Employer Contact	n = 3 (10%)
↓	Other	n = 15 (48%)
↓		
**Employer Agreed to Participate**	**Employer Declined**	
n = 54 (73%)	n = 20 (27%)	
↓		
**Additional Co-Workers Recruited**		
n = 72		

The recruitment then turned to the employers of the eligible and consenting workers. Almost three-quarters of their employers agreed to allow researchers to conduct ergonomic exposure assessments during regular work shifts at their worksite, for a total of 54 injured worker participants. In addition, 72 co-workers were recruited for on-site ergonomic assessments, for a total of 126 participants.

In summary, the recruitment method required a recruitment ratio of about 6.5 potential study subjects for each participating subject (random sample of 822 potential workers from the compensation registry to 126 recruited workers). The greatest loss of potential subjects occurred during initial contact phase by the workers’ compensation agency.

### Recruitment Costs

The average time and costs for contacting and recruiting injured workers, co-workers, and worksites are presented in Table 2. The total time for study recruitment was 845 hours (123 work days) and the cost was CDN$25,945. Recruiting workers from the workers’ compensation registry cost an average of CDN$262 per participating worker, and recruiting their employer and co-workers an average of CDN$240 per worksite.

**Table 2:** Recruitment Costs for Study Participants and Worksites.

Cost Component	Total time (hours)	Estimated cost* per injured worker contact (n = 822 or 189)	Estimated cost* per participating injured worker (n = 54)
Initial contact with injured workers in the compensation registry (n = 822), by workers’ compensation personnel:			
• Telephone calls	274	$ 11.67	$ 177.64
Initial contact with consenting injured workers from compensation registry (n = 189) by researchers:			
• Letters	24	$ 5.87	$ 20.55
• Telephone calls	126	$ 18.33	$ 64.16
	**Total time (hours)**	**Estimated cost** * **per worksite contact (n = 74)**	**Estimated cost** * **per participating worksite (n = 49)**
Recruitment of worksites and co-workers, by researchers:			
• Letters	20	$ 3.10	$ 4.68
• Telephone calls	105	$ 28.33	$ 42.78
• Site visits			
o Transportation	185	$ 97.74	$ 147.61
o Meeting with employers	111	$ 30.00	$ 45.31
**Total Study Recruitment Time and Costs**	845 (123 days)	$262.34 per injured worker participant (n = 54) plus	
		$240.38 per participating worksite (n = 49) including co-workers = **$25,945**	

* in Canadian dollars paid at the time of the study: 2004–2005.

### Representativeness

The mean age of the study sample at the time of recruitment was similar to the mean age of all workers in heavy industry in the province [26] (Table 3). The mean age of the sample was about three years older than the mean age of an injured worker in these heavy industries [30], corresponding to the recruitment of our study participants four to five years after their injury claim.

**Table 3:** Comparison of study sample characteristics to labour force characteristics.

	Study Sample Characteristics	Labour Force Characteristics
Demographics	n = 126 workers	n = 116,268heavy industry workers
• Mean age of employees	41.2 years	42.8 years a
• % of employees who were male	95.2%	84.8% a
Workplace Size	n = 49 study worksites	n = 378,700 provincial worksites
• 1 employee (self-employed)	8.2%	55.7% b
• 2–19 employees	38.7%	38.5% b
• ≥20 employees	53.1%	5.8% b

a = data for heavy industry labour force in British Columbia, 2001 statistics [24].

b = data for all companies in British Columbia, 2006 statistics [23].

The proportion of men in the study sample was somewhat higher than population workforce statistics for heavy industry [26] (Table 3). This is likely due to the fact that our sample was restricted to production workers and/or that the initial contacts were injured workers who are more likely to be male [30].

The proportion of small worksites in the sample (2 to 19 workers) was representative of the worksites in the province as a whole (Table 3). Although we were also successful in recruiting self-employed individuals and workers from larger worksites (20+), they were underrepresented and overrepresented respectively in this sample compared to the distribution of worksites in the province [31].

The 126 participants worked in a broad array of worksites (n = 49), worksite types (n = 29) and occupations (n = 51) (Table 4), representing diverse work environments for exposure assessment.

**Table 4:** Diversity of Worksites and Occupations Represented by 126 Participants in 49 Worksites in 5 Heavy Industries.

Target Industry	Worksite Types	Occupations
Construction n = 23 (18.3%)	Flooring, High Rise Construction, Masonry, Road Paving, Ship Building, Stucco	Asphalt rollerman, Bricklayer, Cabinet maker, Carpenter, Cement finisher, Floor Layer, Iron worker, Labourer, Mason, Plasterer, Plumber, Refurbisher, Supervisor
Forestry n = 24 (19.0%)	Logging, Log Sorting, Saw Repairing, Tree Seed Harvesting	Boomman, Faller, Handyman, Heavy equipment operator, Helicopter pilot, Logging machinery operator, Mechanic, Road builder, Saw filer, Truck driver
Transportation n = 25 (19.8%)	Airline, Box Company, Bus Company, Car Dealership, Disposal Service, Ferry Service, Long Haul Trucking, Package Delivery, Transit Service	Airplane mechanic, Asphalt worker, Automotive mechanic, Bus cleaner, Bus driver, Custodian, Deckhand, Forklift operator, Heavy-duty equipment mechanic, Labourer, Loader operator, Parts clerk, Ramp attendant, Ship engineer, Shipper, Truck driver, Warehouse person
Warehousing n = 30 (23.8%)	Cold Storage, Container Yard, Grain Elevator	Custodian, Dock worker, Forklift operator, Grain elevator operator, Millwright, Parts clerk, Sheet metal worker, Storekeeper, Warehouse person
Wood and wood products n = 24 (19.0%)	Cabinet Manufacturing, Door & Window Manufacturing,Lumber Milling, Paper Making, Staircase Manufacturing,Truss Manufacturing, Wood Turning	Cabinet maker, Fabricator, Forklift operator, Labourer, Log chipper/grinder, Lumber grader, Lumber puller, Paper machine operator, Paper maker, Saw operator, Woodworker

## Discussion

Using injured workers as the point of entry to worksites was a feasible recruitment method. Workers were randomly selected, independent of job position, union affiliation or worksite characteristics. The method resulted in a sample that included workers and worksites that are often the most difficult to recruit and therefore under-represented in conventional recruitment methods: worksites with fewer than 20 employees and self-employed workers (typically trades people at transient construction worksites). The age and sex distribution of the sample indicated that it was representative of the target heavy industry workforce.

The recruitment method was dependent on a list maintained by the workers’ compensation agency. At the time of the study, privacy legislation restricted ‘third party’ contact, so required that the agency obtain permission from each potential participant to release his or her contact information. As a result, agency personnel (who were not researchers) played a key role as the point of first contact [10] and the greatest loss of potential study participants occurred at this point. Other jurisdictions may not face the same legislative challenges. Had the researchers been the first point of contact, they may have been perceived as more neutral and may have been able to respond to concerns about eligibility, time commitment, and the value of the research in a way that improved the recruitment rate. Evidence from a recent survey shows that members of the public look more favorably on research contacts made by university and hospital researchers than those made by government agencies [32]. The added privacy layer was also resource-intensive, necessitating duplication of participant contact, one by the record-holding agency and one by the researchers. The repeated contact may have contributed to ‘annoyance’ as a reason for non-participation.

Recruitment in 2005 and 2006 was based on a random sample of workers with a workers’ compensation claim in 2001. The intent of the lag in recruitment was to identify ‘incident’ cases, for inclusion in epidemiological studies based on work by the investigators related to musculoskeletal (i.e. episodic) disorders [26]. The four-year lag period between claim date and recruitment date contributed to the low contact rate by the workers’ compensation representative. Researchers who choose to use this recruitment method may not need as long a lag period depending upon the health outcome of interest, although some lag time is necessary if the intent is to recruit a workers who are no longer on short or long term work disability, or who have been injury- or illness-free for a period of time.

The recruitment method also included contacting employers to gain access to worksites. It can be difficult to get worksites to agree to participate in occupational research due to concerns about resources, company public image, or interruptions to productivity [4]. We were pleased that 73% of employers agreed to participate despite the unusual route of contact, via their employees. Multiple calls were required to make contact with the employer or supervisor of the consenting worker. Worksite meetings with management were required to establish trust and to get the necessary organizational approvals in place, especially among large employers with more hierarchical structures, as has been reported by others [4].

Using injured workers as the point of entry to worksites was successful in recruiting a representative sample of workers from small employers (those with at least 2 but less than 20 employees), but under-represented the self-employed. This may provide an appropriate recruitment strategy for researchers wanting a population-based sample of workers for epidemiological research given the majority of worksites are small employers [12], [14], while minimizing the ethical and resource challenges of recruiting multiple worksites with a sole employee. The under-representation of the self-employed is likely because these individuals may elect not to have workers’ compensation coverage, are in temporary or precarious employment situations without coverage, or may feel they are unable to take work disability leave as the sole employee [33]. Recruiting temporary, precarious or casual workers is a great challenge not fully solved through workers’ compensation recruitment methods [19], so creative thinking about other novel recruitment approaches is warranted. However, it is important to note that we were able to recruit workers, such as those employed in construction occupations and industries, who are hard to reach because of their employment arrangements (i.e. sub-contracting, self-employed, transient or temporary job sites) [1], [2].

Companies with 20 or more employees were over-represented in our study, in part because they are over-represented in the workers’ compensation statistics and also in part because larger employers are more likely to participate in research. An over-representation of large worksites still affords efficiencies in terms of recruitment and exposure assessment resources for occupational health research.

Labour market restructuring and changes in employment relationships (outsourcing, subcontracting, fragmentation) has resulted in the growth of small businesses in most industrialized or high income countries and these small businesses have different working conditions, exposures and occupational health risks [15], [19], [20], [34], [35]. Younger workers [19] and female workers [36] are also more likely to be found in smaller worksites. In a recent review of studies examining the use of large exposure databases for occupational health research, the number of employees was a significant predictor of exposures [37]. Similarly, a literature review of European Union studies identified higher workplace injury and fatality rates in small worksites and major discrepancies in exposures between small and larger worksites, including greater physical demands/strains with fewer employees [15]. This means that recruiting across worksites with varying workforce sizes should increase the variability of exposures observed and improve the chance of detecting exposure-response relationships. Finally, findings that are representative of small worksites and demonstrate an exposure-response relationship across occupations and industries increase the generalizability of recommendations to prevent/reduce exposures and promote worker health and safety. Small worksites often require a different control approach: ‘upstream’ structures and processes (regulations, policies, industry practices) have the potential for greater impact than addressing conditions or implementing interventions on a workplace-by-workplace basis [20].

Using injured workers as the point of entry to worksites has cost implications as a recruitment method for occupational health research. Information on the costs of occupational health research methods is sparse [21], [24], but is required to make informed decisions about study design and is one of the unique contributions of this paper. The recruitment ratio, i.e., the number of potential subjects for each participating subject, was a major contributor to the cost. In this study, with the potential loss of participants because of privacy legislation requirements and the lag in contact, the ratio was 6.5 potential subjects to 1 participating subject, a significant resource and time commitment. Though high, this ratio may not be as high as that for some study designs. Reports for clinical trials suggest recruitment ratios of 10 to 1 are not atypical [38], [39]. However, it is certainly higher than employer-based studies where the support of the company and union are established prior to worker contact, and participation rates are usually much higher than 50% (recruitment ratios <2). Even though recruitment costs for this method were high, recruitment was still a small proportion of overall study costs (∼7%).

In summary, this recruitment method had a low participation rate and incurred extra costs, but the cost of recruitment remained a small proportion of the study budget. The method achieved a good representation of small worksites, but the self-employed remained under-represented and large workforces over-represented. Compared to common recruitment techniques that focus on large companies, this method achieved more diversity of workforce sizes, a wide array of employers and occupations, and is a worthwhile option to consider for population-based, occupational epidemiological studies of exposure-response relationships.
